# Should I Stay or Should I Go – ERK as Control Lever in Hepatocyte Expansion

**DOI:** 10.1016/j.jcmgh.2025.101532

**Published:** 2025-05-30

**Authors:** Dirk Schmidt-Arras

**Affiliations:** Department of Biosciences and Medical Biology, Paris-Lodron-University, Salzburg, Austria; Center for Tumor Biology and Immunology, Paris-Lodron-University, Salzburg, Austria; Cancer Cluster Salzburg, Salzburg, Austria

The liver is a vital organ with a wide range of functions, including metabolism, detoxification, and immune regulation.^1^ The high regenerative capacity of the liver ensures its central function in the body. In mice, regeneration after two-thirds partial hepatectomy (pHx) restores the original total liver mass within 1 week. Several growth factors and cytokines including hepatocyte growth factor (HGF), epidermal growth factor (EGF), tumor necrosis factor (TNF) α, and interleukin 6 (IL-6) have been shown to be upregulated upon partial hepatectomy.^2^

Lineage tracing has shown that, under physiological conditions, the majority of hepatocytes do not proliferate within a time frame of 9 to 10 months.^3^ However, following acute liver injury, hepatocytes respond by hypertrophy and proliferation.^4^ Wnt signaling, but also EGF, HGF, and IL-6 signaling, have been shown to be essential for regeneration upon acute liver injury.^4^

Mainly produced by Kupffer cells upon exposure to damage or pathogen associated molecular patterns (DAMP/PAMP), IL-6 targets multiple cell types of the liver such as hepatocytes, hepatic stellate cells (HSCs), endothelial cells, and immune cells.

Ex vivo expanded hepatocytes can be used to replace damaged liver in vivo or to be used for in vitro drug testing and research. Efficient large-scale hepatocyte expansion could even represent a replacement for liver organ transplantation. Long-term expansion of differentiated hepatocytes in 2-dimensional (D) cultures has proven difficult. The generation of liver organoids in 3D culture allows long-term cultivation due to the presence of cells with progenitor phenotype.^5^, ^6^ However, the high costs of using organoids for large-scale production of hepatocytes is prohibitive.

In previous work, Xin Xie and colleagues discovered that IL-6, but not TNF, in classical hepatocyte culture medium (HCM containing EGF, HGF, IL-6 or TNF) promoted hepatocyte proliferation in 2D cultures. Albeit reaching a plateau already at 3 ng/mL, only higher concentrations (10–30 ng/mL) supported long-term expansion with over 30 passages. This was linked to the induction of a hepatic progenitor cell (iHPC) phenotype characterized by increased Sox9 and CK19 and decreased albumin expression.^7^ They further demonstrated that long-term culture of these iHPCs did not alter their capacity to differentiate to hepatocytes upon cytokine/growth factor withdrawal and that these cells possess liver repopulating capacity in vivo. Interestingly, Hans Clevers and colleagues made a similar observation. In their experiments, IL-6 promoted initial expansion of adult human hepatocyes into liver organoids.^8^ Initial transcriptomic programs in these hepatocytes were similar to genes expressed in expanding human fetal hepatocytes, despite the age differences of donors. However, continuous growth driven by IL-6 was dependent on restoration of metabolic pathways by FXR activity.

In their current study published in this issue of CMGH, Xin Xie and colleagues investigate in more detail the molecular mechanisms underlying long-term expansion capacity of hepatocytes.^9^ Based on gene expression profiles, they found activation of major signaling pathways such as PI3K-AKT, MAPK, and JAK-STAT pathways. Surprisingly, they also found major hallmarks of TGFβ pathway activation, such as *Tgfb1* expression, Smad2/3 phosphorylation, and induction of mesenchymal genes associated such as *Snai1* and *Vim*. This suggests that TGFβ pathway activation resulted in epithelial to mesenchymal transition (EMT). Induction of TGFβ1 expression and autocrine pathway activation was downstream of HGF-induced MAPK-ERK pathway activation. Inhibition of the TGFβ pathway by small molecules enhanced initial expansion of hepatocytes. Therefore, induction of TGFβ seems to be a negative feedback loop limiting hepatocyte proliferation. Inhibition of TGFβ pathway correlated with the induction of hepatocyte progenitor markers such as CK19, CD24, and CD133 suggesting that TGFβ counterbalances IL-6/STAT3 induced cell fate decisions towards a hepatocyte progenitor phenotype.

Xie and colleagues also showed that both IL-6 and HGF pathways induced activation of the Hippo-YAP pathway either via STAT3 or via MAPK-ERK, which was essential for hepatocyte proliferation. This is in accordance with previous findings that constitutive activation of the IL-6 pathway by activating mutations in the signal transducing IL-6 receptor subunit GP130 activates intestinal epithelial cell proliferation in a STAT3/c-Src dependent manner.^10^

Taken together, the study by Xie and colleagues nicely demonstrates that integration of multiple signalling pathways determines the fate of hepatic epithelial cells. In the future, we need to better understand how different signaling pathways converge at the level of signaling proteins or at gene promotors and enhancers to induce a cooperative effect. This will help to understand cell fate decisions during acute liver injury and to understand how these mechanisms fail in chronic liver disease, and therefore help to design novel therapeutics to boost the regenerative capacity of hepatic epithelial cells in disease and to better understand mechanisms of oncogenic transformation.
